# Transcriptome profiling of *Fraxinus excelsior* genotypes infested by emerald ash borer

**DOI:** 10.1038/s41597-023-02588-z

**Published:** 2023-10-05

**Authors:** James M. Doonan, Chatchai Kosawang, Michael Eisenring, Tim Ladd, Amanda D. Roe, Katharina B. Budde, Hans Jørgen Lyngs Jørgensen, Valentin Queloz, Martin M. Gossner, Lene R. Nielsen

**Affiliations:** 1 Department of Geosciences and Natural Resource Management, University of Copenhagen, Rolighedsvej 23, 1958 Frederiksberg C, Denmark; 2 Forest Health and biotic Interactions, Swiss Federal Research Institute WSL, Zürcherstrasse 111, 8903 Birmensdorf, Switzerland; 3 Great Lakes Forestry Centre, Canadian Forest Service, Natural Resources Canada, Sault Ste. Marie, Ontario Canada; 4 Buesgen Institute of Forest Genetics and Forest Tree Breeding, Georg-August Universität Göttingen, Buesgenweg 2, 37077 Goettingen, Germany; 5 Department of Plant and Environmental Sciences, University of Copenhagen, Thorvaldsensvej 40, 1871 Frederiksberg C, Denmark; 6 Department of Environmental Systems Science, Institute of Terrestrial Ecosystems, ETH Zürich, Universitätstrasse 16, 8092 Zürich, Switzerland

**Keywords:** Forest ecology, Transcriptomics, Biotic

## Abstract

European ash, *Fraxinus excelsior* is facing the double threat of ongoing devastation by the invasive fungal pathogen, *Hymenoscyphus fraxineus* and the imminent arrival of the non-native emerald ash borer (EAB), *Agrilus planipennis*. The spread of EAB which is currently moving westwards from European Russia and Ukraine into central Europe, poses an additional substantial threat to European ash, *F. excelsior*. While the molecular basis for resistance or variation in resistance among European ash genotypes is heavily investigated, comparatively little is known about the molecular ash traits involved in resistance against EAB. In this study we have gathered transcriptomic data from EAB inoculated genotypes of *F. excelsior* that have previously shown different levels of susceptibility to EAB. Resultant datasets show differential gene expression in susceptible and resistant genotypes in response to EAB infestation. This data will provide important information on the molecular basis of resistance to the EAB and allow the development of management plans to combat a pending threat of a culturally and ecologically important European tree species.

## Background & Summary

*Fraxinus excelsior*, known as common or European ash, is a frequent broad-leaf tree species native to Europe. Despite its ubiquity in the area, the species has come under increasing pressure from a devastating fungal disease, known as ash dieback (ADB), which is decimating *F. excelsior* across the European continent since the early 1990s^1,2^. Emerald ash borer (EAB), an invasive alien beetle species in the family Buprestidae, is a new threat for European ash. Like ADB, EAB is native to north-east Asia, where it feeds but poses limited impact on native Asian ash species^3^. EAB was first reported outside its native range in North America in 2002^4^. Currently, six common North American *Fraxinus* species are now either endangered or critically endangered on the IUCN Red list^5^, including *Fraxinus pennsylvanica* (green ash) and *Fraxinus americana* (white ash)^6,7^. This invasive insect has caused substantial ecological and environmental impacts as it killed tens of millions of ash trees in North American countries^4,7^. To date, there is no report of EAB in Central Europe although the beetle has been reported in European Russia and Ukraine^8,9^. In Russia, the beetle has caused a serious decline of *F. pennsylvanica*, which was introduced from North America and local outbreaks of EAB on *F. excelsior* have also been observed recently^10^. With the current known location of EAB at Europe’s eastern border, EAB is predicted to spread into central Europe in the next few years, threatening Europe’s already endangered ash populations^3^. Due to a co-evolution with EAB, the Asian species *Fraxinus mandshurica* generally exhibits greater resistance to EAB than Eastern North American species, including *F. pennsylvanica*, *F. americana*, *Fraxinus quadrangulata* and *Fraxinus nigra*^10–12^. Nevertheless, intraspecific variation in EAB resistance was observed among North American species. For instance, a small number of green ash trees withstood EAB infestations better than others^13^. Recent evidence, mainly from *F. mandshurica* and *F. pennsylvanica*, suggests that resistance to EAB is likely a polygenic trait and several potential genes/proteins in various pathways have been proposed that mediate resistance^14–18^. Interestingly, a set of *F. excelsior* saplings deemed to exhibit high resistance to EAB comparable with that of *F. mandshurica* despite the lack of co-evolution with EAB^19^. In this study, we present transcriptome analysis of four European ash genotypes subjected to EAB infestation under controlled conditions. Two genotypes (B9 and B20) exhibited increased resistance to EAB in a previous experiment, whereas the other two (B3 and B8) were EAB-susceptible (Gossner *et al*.^20^). This study deployed whole-transcriptome sequencing (RNA-Seq) to evaluate the impact of EAB resistance on transcriptional changes in *F. excelsior* genotypes during infestation with EAB. As EAB will inevitably reach Europe, our datasets are valuable resources for research on the biology of EAB resistance in European ash and for guiding breeding for EABresistance.

## Methods

### Ash selection, EAB infestation and sample preparation for NGS

In 2018, four European ash genotypes (B3, B8, B9 and B20) were selected from two plots in Switzerland. Replicates of these genotypes (graftings) were previously exposed to EAB-bioassays and showed contrasting levels of resistance to EAB infestation^20^. To generate replicates of each genotype for the present study, one-year-old, healthy, similar-sized scions were collected from the mother trees in the field in the winter of 2020. Scions were kept on ice in the field and were subsequently stored in a cool room (4 °C) until further processing. All scions were grafted onto common European ash rootstocks (60–80 cm tall) 1–2 weeks after collection. All grafted trees were planted in 4L pots filled with humus-rich soil (Potting soil, Ökohum, Germany) containing 2g long-term fertilizer (18%N, 12%K2O, 6%P2O5 Tardit-Top, Hauert, Switzerland). Trees were kept in outdoor foil tunnels until the beginning of the EAB bioassays. The EAB bioassays are described in detail in Gossner *et al*.^20^. Briefly, EAB eggs for the bioassay were obtained from two insect colonies maintained at the Great Lakes Forestry Centre, Sault Ste. Marie (Glfc:IPQL:AplaPPP01 and Glfc:IPQL:AplaPPP02)^21^. These two families of EAB were initiated from adult insects flushed from green ash log bolts (*F. pennsylvanica*) collected in Presqu’ile Provincial Park, Brighton, Ontario, Canada and reared according to Roe *et al*.^19^.

The import permit was issued by the Federal Office for Agriculture FOAG, Switzerland in 2019 (Letter of authority No. 01 /19) and renewed in 2020 (Letter of authority No. 24/20) and 2021 (Letter of authority No. 36/21). Upon arrival in Switzerland all eggs were transferred to a level 3 biosecurity laboratory in the Plant Protection Lab at WSL (Ecogen nr: A182420) and kept at 25 °C, 55% Relative humidity, 16h Light: 8 h Dark. The eggs were stored for 4–6 days until inoculation for EAB resistance screening. Two weeks prior to EAB infestation the grafted trees were moved from the foil tunnels to biosafety level 3 climate chambers (24 °C, 70% RH, 16 h L: 8h D) at WSL in Switzerland. Trees were infested with EAB during four runs (dates: 12 June–27 July, 14 June-29 July, 19 June-3 August and 29 June- 13 August 2022). In each run 1-2 replicates of each genotype were infested with EAB while a same-sized set of trees remained uninfested (control trees). In total three trees per genotype were inoculated with EAB at two positions on the main stem. EAB eggs were only placed on stem sections that were at least one year old and that were at least 10 cm apart and 10 cm above the graft union. At each inoculation site, four EAB eggs attached to coffee filter strips were directly placed onto the bark and secured with parafilm. Coffee filter strips without EAB-eggs were attached to control trees in a similar fashion. To determine the date of egg-hatching 20–30 EAB eggs were placed in a ventilated Petri dish located next to the experimental trees. Dishes were observed twice a day between 0900–1100 h and 1600–1800 h and the number of freshly hatched EAB larvae were counted. The starting date of a run was defined as the first date on which 50% of all EAB eggs in the dish had hatched. After 45 days all trees were debarked, the EAB larvae were recovered, and the phloem was harvested on dry ice using cold razor blades. All recovered larvae per tree were counted and the dry weight was quantified (Supplementary table 1).Phloem tissue was collected next to beetle galleries in EAB infested trees. Using comparable stem sections phloem tissue was collected from non-infested trees. Harvested phloem was flash-frozen in liquid nitrogen, pulverized with pestle and mortar, and stored at −80 °C.

### RNA isolation and sequencing

**Fig. 1:**
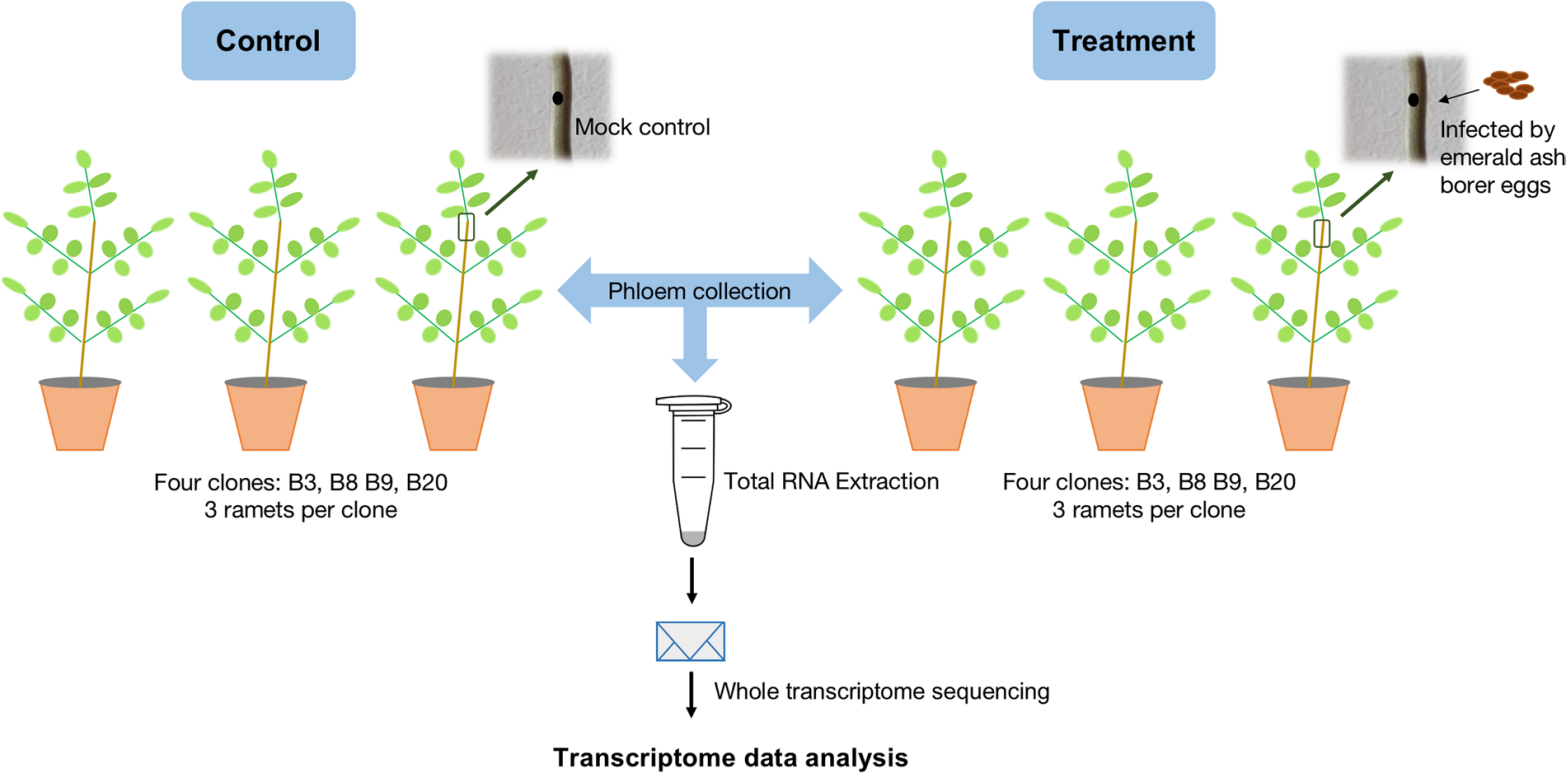
Schematic representation of experimental setup. Ash saplings were divided into control and EAB infested. RNA was extracted from phloem and sequenced.

Total RNA was extracted from phloem samples using the E.Z.N.A Plant RNA Kit (Omega BIO-TEK, USA) according to the manufacturer’s instructions. During the extraction, removal of genomic DNA was carried out simultaneously using the RNase-Free DNase I Set (Omega BIO-TEK, USA). RNA quality and quantity were primarily assessed using a NanoDrop 2000 spectrophotometer (Thermo Scientific, USA) before sending to Macrogen Europe (The Netherlands) for secondary QC, library preparation with poly-A selection and TruSeq stranded mRNA kit (Illumina, USA) and sequencing with Illumina NovaSeq platform with 100 bp paired-end reads. In total, 24 cDNA libraries from two treatments (control and infested with EAB) were constructed for transcriptome sequencing (Fig. 1; Supplementary table 1).

### Data processing and differential gene expression analyses

**Fig. 2:**
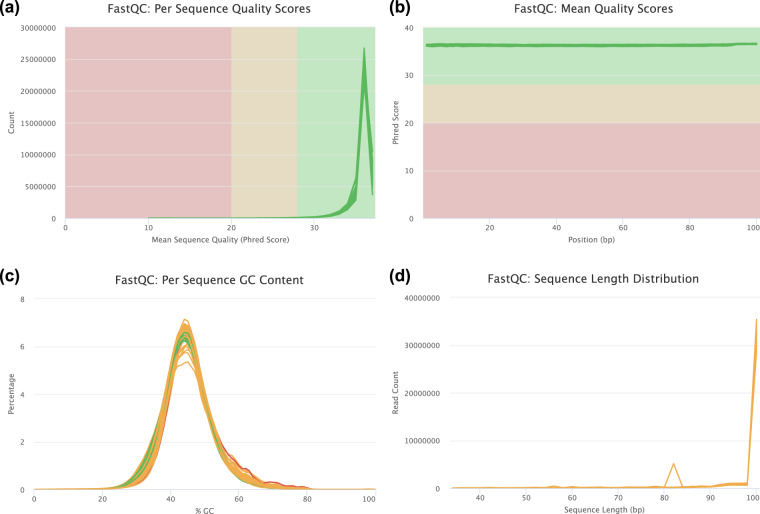
Quality control of sequenced transcripts showing (**a**) sequence quality (Phred score), (**b**) quality score per sequence base position, (**c**) percentage GC content across sequences, (**d**) sequence length distribution per number of transcripts (reads).

The raw reads were quality controlled by removing adapter sequences, low-quality bases (≤Q20) and reads shorter than 35 bases using the BBDuk program from the BBTools suite version 38.90 (Fig. 2)^22^. Ambiguous bases were removed. High quality reads were mapped to *Fraxinus excelsior* BATG-0.5 reference genome^23^ using HISAT v2-2.2.1^24^ with default parameters. We used featureCounts v 2.0.2^25^ from the Subread package and the *F. excelsior* annotation version 4 (TGAC v2) of the BATG-0.5 genome assembly to summarize counts at gene level.

Gene level read count data was compared using the DESeq2 v1.38.3 software package^26^. Read counts from each of the four *F. excelsior* genotypes (B3, B8, B9 and B20) were compared using three control ramets against three EAB inoculated ramets. Therefore, all comparisons were made using six ramets of each genotype, with a 3 × 3 design. Differentially expressed genes (DEGs) were designated as those with a *P* adjusted value <0.05 and fold change (FC) > |2|. P-value distribution is shown in Supplementary figure 1. To reduce noise but preserve large effects within fold change estimates the ‘apeglm’v1.20.0 shrinkage estimator^27^ was applied. The number of differentially expressed genes and direction of regulation (i.e., up or down) for each genotype is presented in Supplementary table 2. To visualise variation within the transcriptome datasets, principal components from normalized read counts were plotted using the ‘plotPCA’ option within DESeq2. Resultant differentially expressed genes were visualised using the EnhancedVolcano software package v1.16^28^. Combined sets of differentially expressed genes were visualised in a Venn diagram using ggvenn v0.1.9^29^ and ggVennDiagram v1.2.2^30^.

## Data Records

Raw transcriptome data of *F. excelsior* genotypes B3, B8, B9 and B20 infested with EAB were deposited at the DDBJ Sequence Read Archive (DRA) under the BioProject PRJDB15336^31^. Count data of *F. excelsior* genotypes B3, B8, B9 and B20 genotypes were deposited in Figshare under a CC-By licence^32^.

## Technical Validation

### Quality control

Low quality and ambiguous bases were trimmed. The quality score of the bases per position were greater than the Phred score of 20 and each read contained a minimum of 35 bases. The GC content of all samples fell within a normal distribution range. These measurements validate the high quality and lack of contaminants in the data. Alignment of the validated reads to the BATG-0.5 *F. excelsior* reference genome gave high mapping rates (minimum 76.31%, maximum 84.58%; Supplementary table 1), ensuring the high quality of data generated in this study. Sequencing output quality was visualized using FastQC^33^ and MultiQC^34^ (Fig. 2). Differentially expressed genes produced by DESeq2 were compared to those produced using EdgeR v3.40.2^35^. The congruence between the programs in each of the four clones was between 60–80%. The code and resultant data for comparative analysis is presented in Figshare^32^.

### Analysis of transcriptome data

**Fig. 3:**
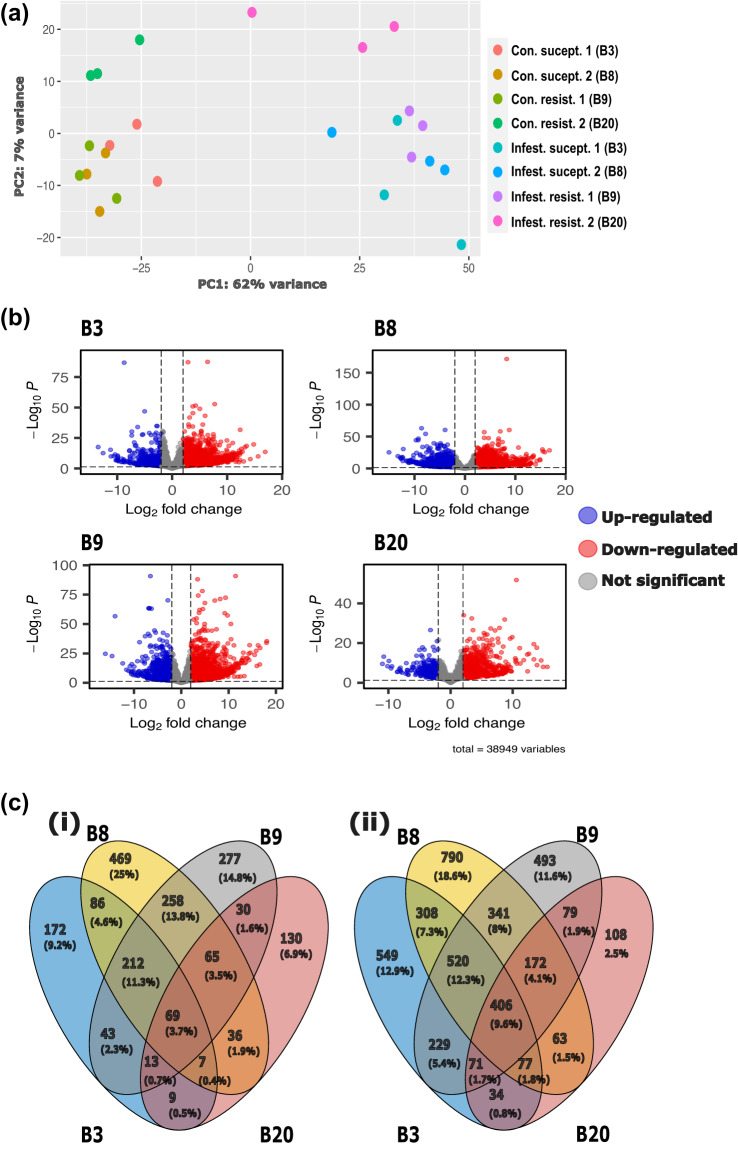
Differential gene expression in EAB susceptible and EAB resistant ash trees. (**a**) Principal component analysis of all biological replicates showing susceptible and resistant control genotypes, and susceptible and resistant EAB infested genotypes. (**b**) Volcano plots showing differentially expressed genes where red indicates significant differential expression in genotype (i) B3, (ii) B8, (iii) B9, and (iv) B20. (**c**) Venn diagrams showing independent and overlapping (i) upregulated genes, and (ii) downregulated genes.

**Fig. 4:**
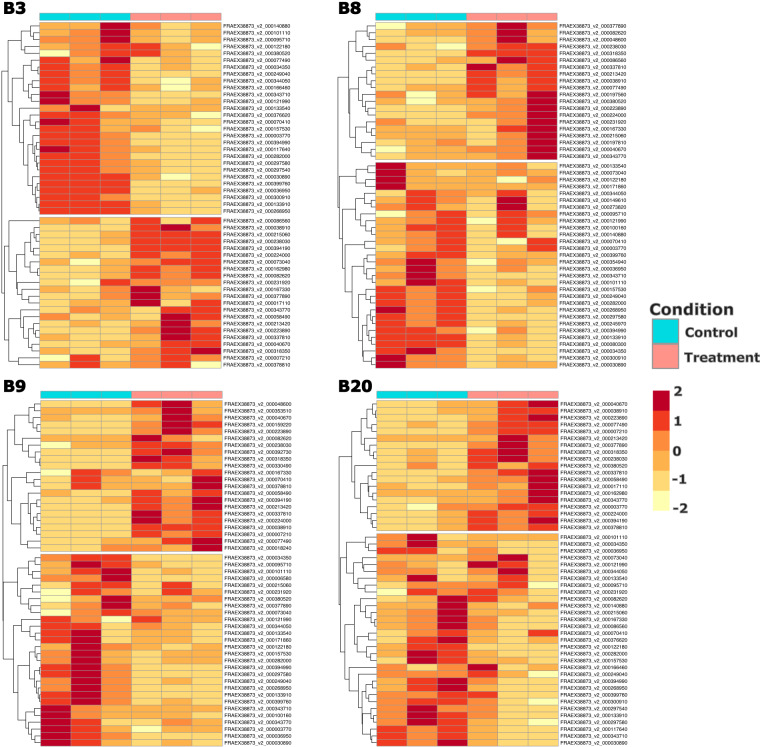
Heatmaps showing the top 50 differentially expressed genes across ramets of each genotype, (i) B3, (ii) B8, (iii) B9, and (iv) B20. N.B. y-axes vary between heat maps.

The read counts of all samples were similar after normalization (Supplementary table 1), which was performed using the internal library size correction methods within DeSeq2. A principal component analysis (PCA) showed clear separation and control trees, with samples from each genotype × infestation treatment combination clustered together (Fig. 3a). Greater dispersion of infested samples compared to control in Fig. 3a may be due to movement of larvae underneath the bark and subsequent difficulty in assigning larvae to infestation sites 45 days post infestation. By comparing the EAB-infested trees with controls, we identified genes that were differentially expressed (DEGs) between each control treatment and EAB infested treatment for each genotype (Fig. 3b-c). Heatmaps show the gene expression pattern of the 50 top ranked DEGs (Fig. 4), where notable DEGs include ethylene responsive transcription factor (FRAEX38873_v2_000164410.1), pathogenesis related protein (FRAEX38873_v2_000394990.1) in clone B3, a plant disease resistance response protein (FRAEX38873_v2_000312240.1) in clone B8, auxin response factor (FRAEX38873_v2_000165740.1) in clone B9 and ethylene responsive transcription factor (FRAEX38873_v2_000007640.1), two pathogen related proteins (FRAEX38873_v2_000146470.1 and FRAEX38873_v2_000308360.1), and a auxin efflux carrier protein (FRAEX38873_v2_000273780.1) in clone B20. The unique responses to EAB infestation in each genotype attest that our data is highly valuable for understanding the biology of resistance to EAB in European ash.

### Supplementary information

Supplementary figure 1

Supplementary information

## Data Availability

All code used in this study is freely available at Figshare 10.6084/m9.figshare.23761401^32^ and GitHub https://github.com/clydeandforth/RNA_seq_EAB.
