# Supplementary figures and images for: Transcriptome profiling of Fraxinus excelsior genotypes infested by emerald ash borer
Source: Sci Data. 2023 Oct 5;10:680. doi: 10.1038/s41597-023-02588-z (PMC10556020; doi:10.1038/s41597-023-02588-z)

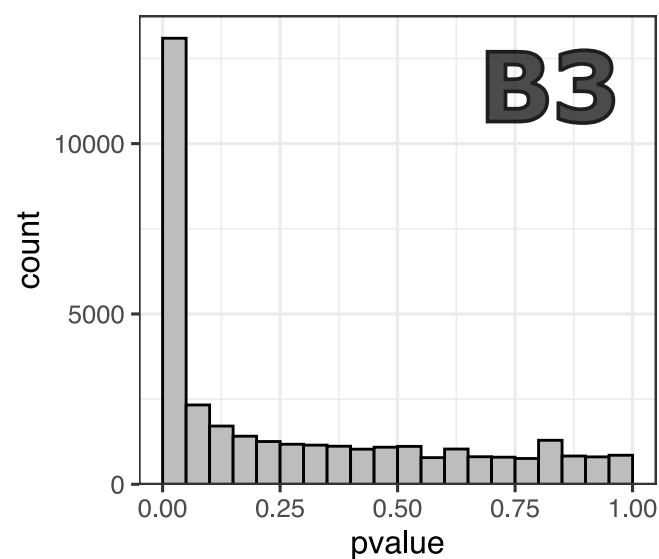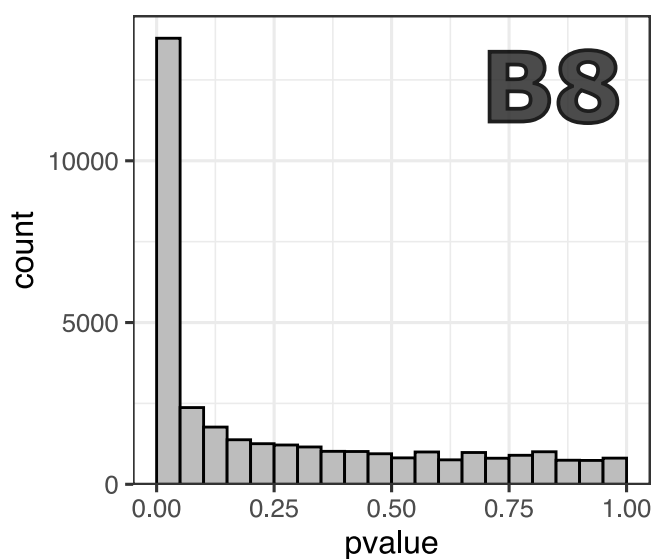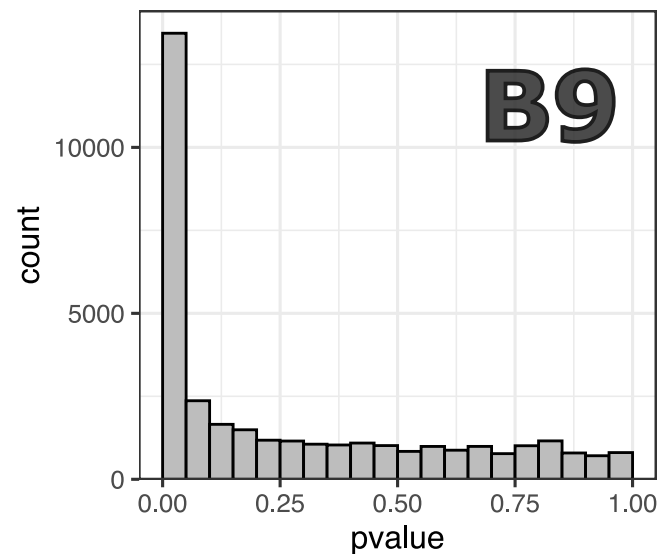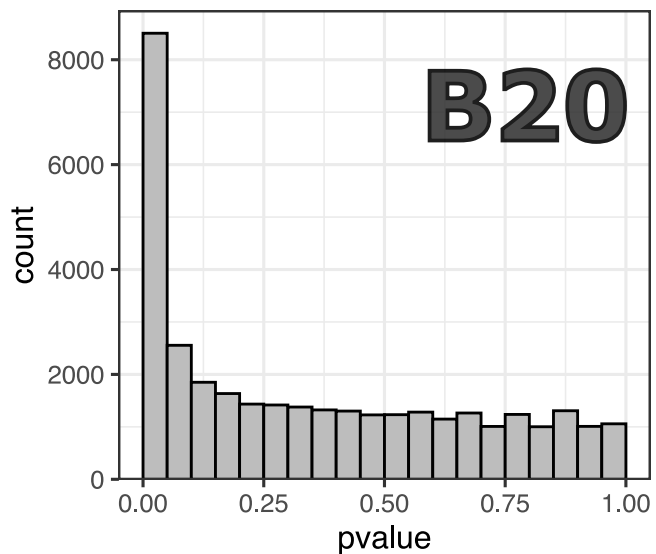

Supplement: Supplementary file 1 — Supplementary figure 1 [file 41597_2023_2588_MOESM1_ESM.pdf]
