# Supplementary material for: Transcriptome profiling of Fraxinus excelsior genotypes infested by emerald ash borer
Source: Sci Data. 2023 Oct 5;10:680. doi: 10.1038/s41597-023-02588-z (PMC10556020; doi:10.1038/s41597-023-02588-z)
Supplement: Supplementary file 2 — Supplementary information [file 41597_2023_2588_MOESM2_ESM.pdf]

Supplementary table 1. Summary of RNA-seq data generated under control treatment and EAB infestations. [1]% of reads that aligned concordantly exactly once.

Sample	Genotype /Provenance	Treatment	EAB resistance	Nr. larvae harvested	Mean dry weight (±SE)	No. of high-quality paired reads	No. of reads and % HISAT2 mapping to BATG-0.5 genome[1]	SRA sample
CH20	B3/Tuggen	Control	NO	-	-	40,032,476	32,839,497 (82.03%)	DRS310129
CH48	B3/Tuggen	Control	NO	-	-	41,350,056	33,230,313 (80.36%)	DRS310130
CH108	B3/Tuggen	Control	NO	-	-	39,572,646	33,212,473 (83.93%)	DRS310131
CH17	B3/Tuggen	EAB	NO	2	19.47±7.83	37,957,108	31,108,723 (81.96%)	DRS310132
CH45	B3/Tuggen	EAB	NO	5	7.45±1.73	39,268,923	32,453,291 (82.64%)	DRS310133
CH105	B3/Tuggen	EAB	NO	5	1.82±0.91	37,119,654	30,568,577 (82.35%)	DRS310134
CH8	B20/Tuggen	Control	YES	-	-	36,004,294	29,973,397 (83.25%)	DRS310135
CH36	B20/Tuggen	Control	YES	-	-	39,327,045	30,012,232 (76.31%)	DRS310136
CH64	B20/Tuggen	Control	YES	-	-	39,612,295	32,736,832 (82.64%)	DRS310137
CH5	B20/Tuggen	EAB	YES	5	0.246±0.09	40,769,175	33,404,826 (81.94%)	DRS31018
CH33	B20/Tuggen	EAB	YES	2	8.15±3.54	42,253,998	33,396,354 (79.04%)	DRS310139
CH125	B20/Tuggen	EAB	YES	3	1.76±1.03	40,189,347	33,111,100 (82.39%)	DRS310140
CH16	B8/Quarten Murg	Control	NO	-	-	35,128,127	29,021,052 (82.61%)	DRS310141
CH44	B8/Quarten Murg	Control	NO	-	-	39,843,272	33,146,514 (83.19%)	DRS310142
CH100	B8/Quarten Murg	Control	NO	-	-	40,120,624	30,621,621 (76.32%)	DRS310143
CH13	B8/Quarten Murg	EAB	NO	5	5.03±1.36	36,227,724	29,389,236 (81.12%)	DRS310144
CH41	B8/Quarten Murg	EAB	NO	4	18.67±7.54	39,265,213	31,428,404 (80.04%)	DRS310145

CH97	B8/Quarten Murg	EAB	NO	4	6.44±1.37	39,780,813	33,644,905 (84.58%)	DRS310146
CH4	B9/Quarten Murg	Control	YES	-	-	39,595,068	32,626,422 (82.40%)	DRS310147
CH32	B9/Quarten Murg	Control	YES	-	-	40,320,822	32,693,802 (81.08%)	DRS310148
CH88	B9/Quarten Murg	Control	YES	-	-	36,193,317	29,988,350 (82.86%)	DRS310149
CH1	B9/Quarten Murg	EAB	YES	3	1.23±0.99	39,296,185	32,096,388 (81.68%)	DRS310150
CH29	B9/Quarten Murg	EAB	YES	5	3.08±0.71	40,376,359	33,159,144 (82.13%)	DRS310151
CH85	B9/Quarten Murg	EAB	YES	3	2.97±0.41	36,797,735	30,126,786 (81.87%)	DRS310152

Supplementary table 2. Differential gene expression between *Fraxinus excelsior* control trees and Emerald Ash Borer infested trees. N.B. upregulated genes are those which are expressed in EAB infested trees & downregulated genes are those which are expressed in control trees. Total expressed genes are the number of unique occurrences of each DEG. Thresholds for differential gene expression; *P* adjusted value less than or equal to 0.05 and log fold change greater than or equal to 2 for upregulated genes and a log fold change less than -2 for downregulated genes. DEGs = differentially expressed genes.

F. excelsior genotype	Number of upregulated genes	Number of downregulated genes	Total number of DEGs
B3	611	2194	2805
B8	2677	1202	3879
B9	2311	967	3278
B20	1010	359	1369
Total expressed genes	4241	1876	6094

Supplementary figure 1. *P*-value distribution of differentially expressed genes in control treatments vs. infested treatments in genotypes B3, B8, B9 and B20.
